# The complete mitochondrial genomes of two vent squat lobsters, *Munidopsis lauensis* and *M. verrilli*: Novel gene arrangements and phylogenetic implications

**DOI:** 10.1002/ece3.5542

**Published:** 2019-09-30

**Authors:** Shao'e Sun, Zhongli Sha, Yanrong Wang

**Affiliations:** ^1^ Deep Sea Research Center Institute of Oceanology Chinese Academy of Science Qingdao China; ^2^ Center for Ocean Mega‐Science Chinese Academy of Sciences Qingdao China; ^3^ Laboratory for Marine Biology and Biotechnology Qingdao National Laboratory for Marine Science and Technology Qingdao China; ^4^ University of Chinese Academy of Sciences Beijing China

**Keywords:** adaptive evolution, Anomura, gene rearrangements, hydrothermal vent, mitochondrial genome

## Abstract

Hydrothermal vents are considered as one of the most extremely harsh environments on the Earth. In this study, the complete mitogenomes of hydrothermal vent squat lobsters, *Munidopsis lauensis* and *M. verrilli*, were determined through Illumina sequencing and compared with other available mitogenomes of anomurans. The mitogenomes of *M. lauensis* (17,483 bp) and *M. verrilli* (17,636 bp) are the largest among all Anomura mitogenomes, while the A+T contents of *M. lauensis* (62.40%) and *M. verrilli* (63.99%) are the lowest. The mitogenomes of *M. lauensis* and *M. verrilli* display novel gene arrangements, which might be the result of three tandem duplication–random loss (tdrl) events from the ancestral pancrustacean pattern. The mitochondrial gene orders of *M. lauensis* and *M. verrilli* shared the most similarities with *S. crosnieri*. The phylogenetic analyses based on both gene order data and nucleotide sequences (PCGs and rRNAs) revealed that the two species were closely related to *Shinkaia crosnieri*. Positive selection analysis revealed that eighteen residues in seven genes (*atp8*, *Cytb*, *nad3*, *nad4*, *nad4l*, *nad5*, *and nad6*) of the hydrothermal vent anomurans were positively selected sites.

## Graphical abstract

The mitogenomes of *M. lauensis* and *M. verrilli* show novel arrangements of mitochondrial genes. Comparisons of mitochondrial gene rearrangements, to some extent, are a useful tool for phylogenetic studies.

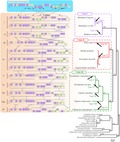

## 1 INTRODUCTION

The Anomura MacLeay, 1838 is a highly diverse infraorder of decapod, including seven superfamilies, 17 families, and approximately 2,500 species (Ahyong, Schnabel, & Maas, 2009; Bracken‐Grissom, Cannon, Cabezas, Feldmann, & Crandall, 2013; Schnabel, Ahyong, & Maas, 2011). The Galatheoidea are the most diverse superfamily within Anomura, with over 1,200 species placed in 69 genera, and have adapted to a wide range of habitats in freshwater, terrestrial, shallow‐water coral reefs, and hydrothermal vent ecosystems (Baba et al., 2008; De Grave et al., 2009). Deep‐sea hydrothermal vent is one of the chemosynthetically driven ecosystems and characterized with high temperature (up to 390°C), low oxygen levels, enriched hydrogen sulfide (H2S), methane (CH4), and heavy metals, such as iron, zinc, and copper (Little & Vrijenhoek, 2003). Decapod crustaceans, such as alvinocaridid shrimps, bythograeid crabs, and galatheid squat lobsters, are dominant fauna in the hydrothermal vents, representing approximately 10% of all taxa reported from these vents (Little & Vrijenhoek, 2003; Martin & Haney, 2005; Yang et al., 2013). Recently, the hydrothermal vent bythograeid crabs (Hui, Song, Liu, Li, & Cui, 2017) and alvinocaridid shrimps (Cottin et al., 2010; Hui, Cheng, & Sha, 2018; Wang et al., 2017; Zhang, Sun, Luan, Lian, & Sun, 2017) have demonstrated numerous genetic basis for the adaptations to vent habitats. However, little genomic and molecular genetic information are available for hydrothermal vent galatheid squat lobsters, impeding the study for the molecular mechanism in their adaptation process. A powerful system is needed to examine the adaptation evolution at the molecular level (e.g., mitochondrial genome).

The metazoan mitochondrial genome (mitogenome) is typically a circular double strand DNA molecule, encoding 13 protein‐coding (PCG) genes (seven subunits of the NADH dehydrogenase complex, the cytochrome b subunit of the cytochrome bc1 complex, three subunits of the cytochrome c oxidase, and two subunits of ATP synthase), 22 transfer RNAs (tRNA) genes, two ribosomal RNAs (rRNA, *rrnS*, and *rrnL*) genes, and a control region (CR) including sites for the initiation of transcription and replication (Boore, 1999). Owing to its small genome size, higher evolutionary rates, limited recombination, and maternal inheritance, (Gissi, Iannelli, & Pesole, 2008; Simon, Buckley, Frati, Stewart, & Beckenbach, 2006), mitogenome has been widely used in species identification (Fu, Han, & Xiao, 2014; Kanmiya et al., 2011), molecular evolution (Cameron, 2014; Shao et al., 2015; Shao, Zhu, Barker, & Herd, 2012), phylogenetic relationship (Cameron, 2014; Cameron, Yoshizawa, Mizukoshi, Whiting, & Johnson, 2011; Chen, Wei, Shao, Dou, & Wang, 2014; Chen, Wei, Shao, Shi, et al., 2014), and population genetic (Wei et al., 2012; Zhang et al., 2014) studies. Although the gene content is relatively conservative, their rearrangements have been frequently reported, particularly in invertebrates at many taxonomic levels (Cameron, Johnson, & Whiting, 2007; Hassanin, Léger, & Deutsch, 2005). The gene rearrangement within a lineage has been supposed to be phylogenetically informative; therefore, comparative analysis of mitochondrial gene order has been proved to be a valuable phylogenetic tool (Akasaki et al., 2006; Boore & Brown, 1998; Smith, Arndt, Gorski, & Fajber, 1993; Yang, Ye, & Huang, 2016; Yuan, Li, Yu, & Kong, 2012). Based on the comparative analysis of mitochondrial gene arrangement, Smith et al. (1993) suggest that the sea cucumbers should group with sea urchins and sea stars with brittle stars. Akasaki et al. (2006) examined the mitochondrial gene arrangements of subclass Coleoidea and claimed that Octopoda showed the ancestral gene order, and the arrangements of mitochondrial genes in Oegopsida and Sepiida were derived from those of Octopoda. Based on the study of gene order rearrangements and phylogenetic relationships of five species belonging to Tellinoidea, Yuan et al. (2012) prefer to put the genus Sinonovacula within the superfamily Solenoidea instead of the superfamily Tellinoidea. Extensive mitochondrial gene rearrangements have been observed in crustaceans, such as copepods, anomuran, and brachyuran decapods, among which more frequent gene rearrangements exhibit compared with the putative ancestral gene order (Ki, Park, & Lee, 2009; Kim, Choi, Park, & Min, 2013; Machida, Miya, Nishida, & Nishida, 2002).

The 13 PCGs of mitogenome are all key subunits of complexes directly involved in the oxidative phosphorylation (OXPHOS) process, directly providing 95% free energy for cells, which is important for metabolic demands in organisms (Gu et al., 2016; Wu, Gu, Guo, Huang, & Yang, 2016). In recent years, the mitogenome has become a powerful system for examining the genetic basis of organismal adaptation to various harsh environments, and signals of positive selection have been detected in mitochondrial genes of various taxa (Korkmaz, Aydemir, Temel, Budak, & Başıbüyük, 2017; Luo, Yang, & Gao, 2013; Scott et al., 2011; Wang et al., 2016; Yu, Wang, Ting, & Zhang, 2011; Yuan et al., 2018; Zhang et al., 2017; Zhou, Shen, Irwin, Shen, & Zhang, 2014). Most of these studies focused their attention on vertebrates, whereas few reports examined the adaptive evolution of crustacean mitogenomes to hydrothermal vent environments (Sun, Hui, Wang, & Sha, 2018; Wang et al., 2017). The molecular evolution of mitochondrial protein‐coding genes in hydrothermal vent squat lobsters are still poorly understood. The mitogenome resources for the Anomura are limited to only ten mitogenomes as recorded on GenBank thus far, with five species from hydrothermal vents (http://blast.ncbi.nlm.nih.gov).

The *Munidopsis* is the second largest genus of galatheid squat lobsters, after *Munida*, with over 200 species, among which ten are endemic to the hydrothermal vent environments (Baba et al., 2008; Martin & Haney, 2005). In this study, we newly sequenced and annotated two complete mitogenomes of the hydrothermal vent squat lobsters, *M. lauensis* and *M. verrilli*. Combined with ten available anomuran mitogenomes, we performed a comparative mitogenomics analysis, in order to: (a) investigate the characteristics of Anomura mitogenomes; (b) assess the phylogenetic information of mitochondrial gene rearrangements; (c) rebuild a mitochondrial phylogeny of the Anomura that could be used as framework for further evolutionary studies; and (d) detect the signals of positive selection of mitochondrial genes in hydrothermal vent anomuran species during their adaptation to deep‐sea hydrothermal vent environments.

## 2 METHODS AND METHODS

### 2.1 Sampling and DNA extraction

The hydrothermal vent squat lobsters, *M. lauensis* and *M. verrilli*, were captured from hydrothermal vent chimney at a depth of 1,121.5 m (119°17′08.321″E; 22°06′55.526″N) and 1,198.7 m (119°17′08.079″E; 22°06′55.432″N) in southwest Pacific Ocean, respectively. Both specimens were collected using the remotely operated vehicle (ROV) Quasar MkII of SMD in the United Kingdom, which was deployed using the RV KEXUE. They were immediately preserved in 95% ethanol after taken until DNA extraction. Total genomic DNA was extracted using the DNeasy tissue kit (Qiagen) accordingly.

### 2.2 Illumina sequencing, genome assembly, and annotation

NEBNext® Ultra™ DNA Library Prep Kit for Illumina (NEB) was used to generate the sequencing libraries following manufacturer's instructions. And then, the index codes were added to attribute sequences to the sample. The clustering of the index‐coded sample was performed on a cBot Cluster Generation System. Sequencing was performed based on an Illumina HiSeq 2500 platform, with the paired‐end reads generated for each sample. The paired‐end raw reads were filtered, and the reads with average quality value lower than Q20 were excluded from further analysis (Sun, Hui, Wang, et al., 2018; Sun, Sha, & Wang, 2018a). CLC Genomics Workbench v. 11.0.64 (http://www.clcbio.com/products/clcgenomics-workbench/) and SOAP denovo (k‐mer = 55) (Li et al., 2010) were selected to assemble the clean data. De novo assembled contigs longer than 10 Kbp were blasted against the NCBI nr database using the “BLAST” tool implemented in the CLC Genomics Workbench to extract the “mitochondrial DNA” contigs. The cutoff E‐value of 1.0E‐15 was used. In order to identify contigs of mitochondrial origin, we aligned the putative mtDNAs of *M. lauensis* and *M. verrilli* with the published complete mitochondrial genomes of the Galatheoidea, *Kiwa tyleri* (KY423514), *Munida gregaria* (KU521508), *Neopetrolisthes maculatus* (KC107816), *Shinkaia crosnieri* (EU420129), and *Petrolisthes haswelli* (LN624374) with the aid of “Alignment” tool implemented in the CLC Genomic Workbench with the default setting. In order to establish a circular mitochondrial DNA (mtDNA), the contigs identified as mitogenome sequences were manually checked for overlap at the beginning and end of the sequence. To evaluate the average sequence coverage of mitochondrial genomes, we mapped sequences against the assembled mitochondrial genomes using GNUMAP (Clement et al., 2010).

The protein‐coding genes were searched by ORF Finder (http://www.ncbi.nlm.nih.gov/gorf/gorf.html), BLASTx, and MITOS Web Server (Bernt et al., 2013) using the invertebrate mitochondrial genetic code. The sequences and positions of tRNA genes were determined by ARWEN (Laslett & Canback, 2008) and MITOS Web Server (Bernt et al., 2013) with the default search mode. The rRNA genes were identified by blasting the inferred sequences against to other published crustacean mtDNA sequences (http://www.ncbi.nlm.nih.gov/BLAST). The gene maps of the *M. lauensis* and *M. verrilli* mitogenomes were drawn with the program CGView (Stothard & Wishart, 2005). The complete mtDNA sequences of *M. lauensis* and *M. verrilli* have been deposited in the GenBank database with the accession numbers MH717895 and MH717896, respectively.

### 2.3 Sequence analysis

The relative synonymous codon usage (RSCU) values and nucleotide composition were calculated using MEGA 5 (Tamura et al., 2011). The GC and AT‐skew values were obtained according to the formulae by Perna and Kocher (1995): AT‐skew = (A−T)/(A+T); GC‐skew = (G−C)/(G+C), where A, T, G, and C are the occurrences of the four nucleotides. DnaSP5.1 (Librado & Rozas, 2009) was taken to determine the effective number of codons (ENC) and the codon bias index (CBI) for each PCG. Tandem Repeats Finder 4.0 (Benson, 1999) was used to search the tandem repeat sequences, and the potential secondary structures of the repeat sequences were predicted by Mfold software version 3.2 (Zuker, 2003). When more than one secondary structures were detected, the most stable one with lowest free energy △G was selected.

### 2.4 Build phylogeny from gene order data

Along with mitogenome sequences of *M. lauensis* and *M. verrilli* (this study), other 10 available mitogenomes from Anomura, including *Paralithodes brevipes* (AB735677), *Petrolisthes haswelli* (LN624374), *Pagurus longicarpus* (AF150756), *Paralithodes camtschaticus* (JX944381), *Lithodes nintokuae* (AB769476), *Clibanarius infraspinatus* (LN626968), *K. tyleri* (KY423514), *M. gregaria* (KU521508), *N. maculatus* (KC107816), and *S. crosnieri* (EU420129), were used in gene order comparison. CREx (Bernt et al., 2007) was used to conduct pairwise comparisons of the mitochondrial gene order. CREx inferred the most possible scenarios for gene rearrangements based on common intervals. MLGO web server (http://www.geneorder.org/server.php; Hu, Lin, & Tang, 2014; Zhou, Lin, Feng, Zhao, & Tang, 2017) was used to infer a phylogeny from gene order data.

### 2.5 Build phylogeny from nucleotide sequences

Neighbor‐joining (NJ) tree based on uncorrected *p* distances among mitochondrial tRNA genes from 12 Anomura taxa (described above) was constructed using MEGA 5 (Tamura et al., 2011). Maximum likelihood (ML) and Bayesian inference (BI) were employed for phylogenetic reconstructions of the 12 Anomura species based on nucleotide sequences of 13 PCGs and 2 rRNA genes using 14 species from five other decapod infraorders (Table S1) as outgroup taxa. The nucleotide sequences for the PCG and rRNA genes were aligned with MAFFT version 6 online (http://mafft.cbrc.jp/alignment/software/), applying the E‐INS‐I manual strategy with default parameters. Areas of dubious alignment were recognized by the program Gblocks (Talavera & Castresana, 2007) (default setting) and excluded from the analyses. PartitionFinder v1.1.1 (Lanfear, Calcott, Ho, & Guindon, 2012) was used to determine the best partitioning schemes and corresponding substitution models. The data blocks were predefined by genes and codon positions for nucleotide sequences of protein‐coding genes. The Bayesian information criterion (BIC) and the greedy heuristic search algorithm with branch lengths were estimated as “unlinked” to identify the best‐fit partition schemes. The best‐fit partitioning schemes (Table S2) were adopted in the phylogenetic analyses.

Maximum likelihood was employed in RAxML Black‐Box webserver (http://phylobench.vital-it.ch/raxml-bb/index.php; Stamatakis, Hoover, & Rougemont, 2008). Bootstrap (BP) values were determined using 1,000 bootstrap replicates. BI analysis was performed by MrBayes 3.1 software (Ronquist & Huelsenbeck, 2003). The Markov chain Monte Carlo (MCMC) was run for 10,000,000 generations (sampling every 1,000 generations) to allow adequate time for convergence. When the standard deviation of split frequencies was <0.01, the run was stopped. All parameters were checked with Tracer v 1.5 (Drummond & Rambaut, 2007). After omitting the first 5,000 “burn in” trees, the remaining 5,000 sampled trees were selected to estimate the 50% majority rule consensus tree and the Bayesian posterior probabilities (PP).

### 2.6 Determine the signals of selection

The codon‐based likelihood approach implemented in the CODEML program from PAML (Yang, 2007) was used to evaluate the potential selective pressures in the mitochondrial PCGs of hydrothermal vent anomurans. The 13 individual PCGs and the concatenated dataset were involved in the positive selection analysis. The tree topologies inferred from tree‐building methods in the present study were used. The ratio of nonsynonymous to synonymous substitution rates (Ka/Ks, denoted *ω*) was taken as a measure of selective pressure. The signals of selection were assessed under several models: one‐ratio model (M0), free‐ratio model (M1), and two‐ratio model (M2). To identify the probabilities of specific residues under positive selection in each gene of the hydrothermal vent anomurans species (marked as foreground branch), the branch‐site Model A (positive selection model) was selected, which allowed *ω* to vary across lineages and sites. All the positively selected sites were determined by Bayes empirical Bayes (BEB) method (Yang, Wong, & Nielsen, 2005) with posterior probabilities of ≥0.95.

## 3 RESULTS AND DISCUSSION

### 3.1 De novo assemblies of *M. lauensis* and *M. verrilli* mitogenomes

The Hiseq runs resulted in 33,862,831 (10.16 G) and 46,095,676 (13.83 G) paired‐end clean reads from *M. lauensis* and *M. verrilli* libraries, respectively. The sequencing qualities were generally high for both squat lobsters. About 93.77% of the reads in *M. lauensis* and 90.54% of the reads in *M. verrilli* passed Q20, indicating the probability of a base call error ≤0.01. There were in total 425,589 and 579,932 contigs assembled de novo based on the paired‐end reads for *M. lauensis* and *M. verrilli*, respectively. The lengths of most contigs (82.1% and 85.3% in *M. lauensis* and *M. verrilli*, respectively) were <1 Kbp. Only eleven *M. lauensis* contigs and thirteen *M. verrilli* contig had lengths longer than 10 Kbp. The average sequence coverage was 11.0 and 13.0 for all the assembled contigs of *M. lauensis* and *M. verrilli*. The blast results suggested that the top hits (*E*‐value = 0) of the longest contig in each sample (17,520 and 17,659 bp for *M. lauensis* and *M. verrilli*, respectively) were the mitogenomes of Galatheoidea species. Therefore, there was a highly possibility that the longest contig in each sample was the mitogenome of *M. lauensis* or *M. verrilli*, which was assembled from multiple overlapping reads. A total of 29,861 (*M. lauensis*) and 40,648 (*M. verrilli*) multiple overlapping reads were mapped onto the longest mitochondrial contigs, giving an average coverage 511× for *M. lauensis* and 691× for *M. verrilli* mtDNAs, which were about 46–53 times higher than that of all contigs. The higher sequencing coverage of mtDNAs is consistent with the high copy numbers of mitochondria in eukaryotic cells and indirectly confirm the mitochondrial origin of the sequences (Hung et al., 2013).

### 3.2 General genome characteristics

**Figure 1:**
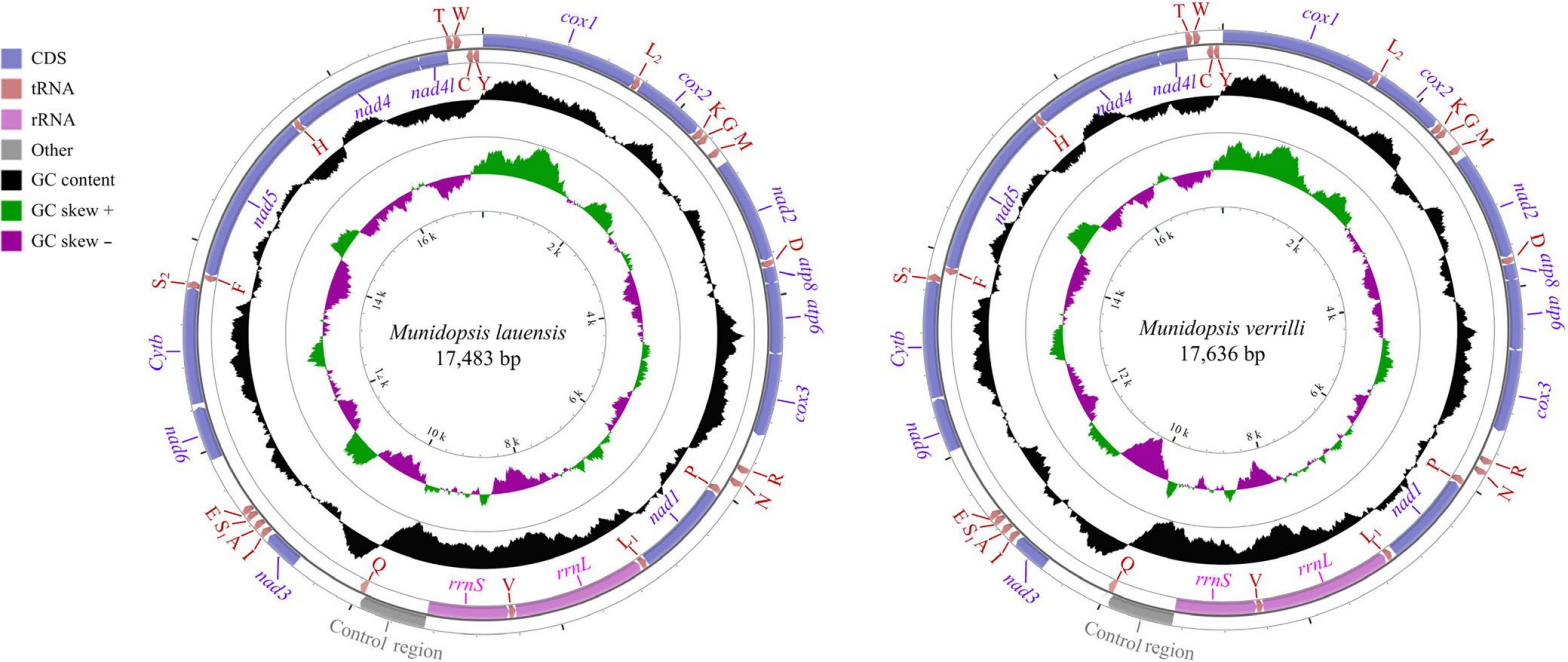
The organization of the mitogenomes of *Munidopsis lauensis and M. verrilli*. The full names of protein‐coding genes, *rrnS* and *rrnL,* are listed under abbreviations. *rrnS* and *rrnL*, 12S and 16S ribosomal RNA genes, respectively; *atp6* and *atp*8, ATPase subunit 6 and 8 genes, respectively; *cox1‐cox3*, cytochrome c oxidase subunits I‐III genes, respectively; *cytb*, cytochrome b gene; *nad1‐6* and *4l*, NADH dehydrogenase subunit 1–6 and 4 L genes, respectively. One uppercase letter amino acid abbreviations are used to label the corresponding tRNA genes

The complete mitogenomes of *M. lauensis* and *M. verrilli* were 17,483 bp and 17,636 bp in length, respectively (Figure 1, Table 1). The sizes of both mitogenomes are the largest among the length range of all available Anomura mitogenomes (approximately 16,000 bp). The plausible explanation for this phenomenon may be the extension of noncoding regions, which were 2,077 and 2,200 bp in *M. lauensis* and *M. verrilli*, respectively. Each genome contained the typical 13 PCGs, 22 tRNA genes, 2 rRNA genes, and one control region (CR). Within these genes, 9 PCGs and 14 tRNAs were encoded by the light strand, while 4 PCGs, 8 tRNAs, and 2 rRNAs were encoded by the minority strand. Considering their location and AT‐richness, we supposed continuous region between *rrnS* and *trnQ* to be the CR as in the case of the hydrothermal vent galatheid crab *S. crosnieri* (Yang & Yang, 2008). The overlapping nucleotides from seven adjacent genes in the mitogenome of *M. lauensis* were discovered up to 27 bp in total. In the case of *M. verrilli* mitogenome, eight overlaps between adjacent genes were up to 28 bp.

**Table 1:** Organization of the mitogenomes of *Munidopsis lauensis* and *M. verrilli*

Feature	Position		Length (stop codon included)		Start codon		Stop codon		Anticodon	Strand
	*Ml* (Intergenic nucleotides)^a^	*Mv* (Intergenic nucleotides)^a^	*Ml*	*Mv*	*Ml*	*Mv*	*Ml*	*Mv*		
*cox1*	1–1,503 (−6)	1–1,503 (−4)	1,503	1,503	ATT	ATT	TAA	TAA		+
*trnL2*	1,498–1,566 (6)	1,500–1,564 (8)	69	65					TAA	+
*cox2*	1,573–2,253 (8)	1,573–2,253 (11)	681	681	ATG	ATG	TAA	TAA		+
*trnK*	2,262–2,331 (2)	2,265–2,333 (2)	70	69					TTT	+
*trnG*	2,334–2,396 (103)	2,336–2,398 (138)	63	63					TCC	+
*trnM*	2,500–2,567 (87)	2,537–2,604 (61)	68	68					CAT	+
*nad2*	2,655–3,659 (6)	2,666–3,703 (6)	1,005	1,038	ATT	ATT	TAA	TAA		+
*trnD*	3,666–3,732 (0)	3,710–3,777 (0)	67	68					GTC	+
*atp8*	3,733–3,891 (−7)	3,778–3,936 (−7)	159	159	GTG	GTG	TAA	TAA		+
*atp6*	3,885–4,559 (−1)	3,930–4,604 (−1)	675	675	ATG	ATG	TAA	TAA		+
*cox3*	4,559–5,350 (326)	4,604–5,395 (281)	792	792	ATG	ATG	TAA	TAA		+
*trnR*	5,677–5,744 (74)	5,677–5,743 (45)	68	67					TCG	+
*trnN*	5,819–5,884 (69)	5,789–5,854 (95)	66	66					GTT	+
*trnP*	5,954–6,026 (8)	5,950–6,022 (9)	73	73					TGG	−
*nad1*	6,035–7,000 (2)	6,032–6,973 (26)	966	942	GTG	GTG	TAG	TAA		−
*trnL1*	7,003–7,070 (8)	7,000–7,065 (11)	68	66					TAG	−
*rrnL*	7,079–8,410 (2)	7,077–8,409 (0)	1,332	1,333						−
*trnV*	8,413–8,478 (0)	8,410–8,475 (−1)	66	66					TAC	−
*rrnS*	8,479–9,291 (0)	8,475–9,297 (0)	813	823						−
*CR*	9,292–9,941 (0)	9,298–9,944 (0)	650	647						−
*trnQ*	9,942–10,008 (644)	9,945–10,011 (628)	67	67					TTG	+
*nad3*	10,653–11,006 (26)	10,640–10,993 (24)	354	354	ATT	ATT	TAA	TAA		+
*trnI*	11,033–11,099 (30)	11,018–11,084 (32)	67	67					GAT	+
*trnA*	11,130–11,196 (40)	11,117–11,184 (42)	67	68					TGC	+
*trnS1*	11,237–11,301 (0)	11,227–11,291 (0)	65	65					TCT	+
*trnE*	11,302–11,370 (531)	11,292–11,359 (691)	69	68					TTC	+
*nad6*	11,902–12,417 (32)	12,051–12,566 (11)	516	516	ATC	ATC	TAA	TAA		+
*cytb*	12,450–13,554 (−4)	12,578–13,703 (−3)	1,105	1,126	ATT	ATC	T ‐ ‐	T ‐ ‐		+
*trnS2*	13,551–13,621 (16)	13,701–13,769 (15)	71	69					TGA	−
*trnF*	13,638–13,701 (1)	13,785–13,848 (−3)	64	64					GAA	−
*nad5*	13,702–15,430 (0)	13,846–15,577 (0)	1,729	1,732	ATG	ATG	T ‐ ‐	T ‐ ‐		−
*trnH*	15,431–15,498 (−1)	15,578–15,643 (−2)	68	66					GTG	−
*nad4*	15,498–16,837 (−7)	15,643–16,982 (−7)	1,340	1,340	ATG	ATG	TA ‐	TA ‐		−
*nad4l*	16,831–17,133 (1)	16,976–17,278 (1)	303	303	ATG	ATG	TAA	TAA		+
*trnT*	17,135–17,202 (5)	17,280–17,347 (5)	68	68					TGT	+
*trnW*	17,208–17,277 (39)	17,353–17,422 (49)	70	70					TCA	−
*trnC*	17,317–17,381 (−1)	17,472–17,536 (0)	65	65					GCA	−
*trnY*	17,381–17,445 (38)	17,537–17,599 (37)	65	63					GTA	−

a Intergenic regions refer to noncoding bases between the feature on the same line and the feature on the above line, with a negative number indicating an overlap.

The base composition (A+T content, G+C content) and strand asymmetry (AT‐skew, GC‐skew) were usually used to investigate the nucleotide‐compositional behavior of mitogenomes (Hassanin et al., 2005). The nucleotide compositions of the complete mtDNA sequence for *M. lauensis* and *M. verrilli* were both biased toward A and T (Table 2). The A+T content was 62.40% in *M. lauensis* and 63.99% in *M. verrilli*, which were the lowest among the available Anomura mitogenomes. The lowest A+T content was also found in the PCGs, tRNAs, and rRNAs (Table 2). In order to further evaluate the base bias in the mitogenomes, we measured skewness in different gene regions of *M. lauensis* and *M. verrilli* mitogenomes, and found the whole genomes of the hydrothermal vent squat lobsters were all positively AT‐skewed (0.086 and 0.077) and negatively GC‐skewed (−0.336 and −0.363). The AT‐skew and GC‐skew of the two mitogenomes were all stronger than those of the other anomurans (Table 2).

**Table 2:** Genomic features of the mitogenomes of Anomura species

Species	Genome				13 Protein‐coding genes			rRNAs			tRNAs			Control region		
	Length (bp)	AT%	AT‐Skew	GC‐Skew	AT%	AT‐Skew	GC‐Skew	AT%	AT‐Skew	GC‐Skew	AT%	AT‐Skew	GC‐Skew	AT%	AT‐Skew	GC‐Skew
*Paralithodes camtschaticus*	16,720	73.86	0.003	−0.132	71.57	−0.168	0.014	77.98	0.038	0.238	75.12	0.008	0.146	–	–	–
*Lithodes nintokuae*	15,731	73.28	−0.003	−0.127	71.28	−0.176	0.031	77.97	0.041	0.235	76.78	0.024	0.110	–	–	–
*Paralithodes brevipes*	16,303	72.50	0.009	−0.134	70.23	−0.173	0.034	77.42	0.039	0.240	75.99	0.020	0.156	–	–	–
*Petrolisthes haswelli*	15,348	70.01	−0.019	−0.244	68.61	−0.194	−0.011	73.18	0.035	0.302	72.15	−0.015	0.136	76.45	−0.041	−0.324
*Pagurus longicarpus*	15,630	71.28	0.029	−0.213	69.61	−0.170	−0.013	77.15	0.011	0.310	73.17	0.024	0.126	–	–	–
*Clibanarius infraspinatus*	16,504	67.94	0.042	−0.199	66.37	−0.193	0.037	71.18	−0.050	0.322	70.62	0.001	0.142	69.47	−0.023	−0.118
*Neopetrolisthes maculatus*	15,324	71.26	−0.020	−0.210	70.13	−0.185	−0.005	74.71	0.080	0.294	72.71	0.011	0.091	75.14	0.002	−0.118
*Kiwa tyleri*	16,865	79.32	−0.044	−0.220	76.18	−0.176	0.005	83.64	0.067	0.039	78.92	0.030	0.062	–	–	–
*Munida gregaria*	16,326	74.94	−0.020	−0.162	72.47	−0.196	0.051	79.26	−0.015	0.335	76.12	0.001	0.140	84.95	−0.013	−0.131
*Shinkaia crosnieri*	15,182	72.88	−0.014	−0.313	70.96	−0.184	−0.025	77.92	0.045	0.336	74.46	0.006	0.121	83.49	−0.158	−0.741
*Munidopsis lauensis*	17,483	62.40	0.086	−0.336	60.13	−0.180	−0.034	70.49	−0.015	0.384	69.59	0.023	−0.224	73.38	0.392	−0.283
*Munidopsis verrilli*	17,636	63.99	0.077	−0.363	61.64	−0.184	−0.039	70.83	0.002	0.386	69.25	0.020	−0.236	75.27	0.228	−0.313

### 3.3 Protein‐coding genes and codon usage

In the mitogenomes of *M. lauensis* and *M. verrilli*, the region of PCGs was 11,128 and 11,161 bp in size (stop codon included), respectively. And the overall A+T content of the 13 PCGs was 60.16 (*M. lauensis*) and 61.64% (*M. verrilli*), which were lower than those of other anomurans. The AT‐skew and GC‐skew of the PCGs in both mitogenomes were negative (Table 2). In the mitogenomes of *M. lauensis* and *M. verrilli*, 11 PCGs began with the standard ATN start codon. The codon GTG was found to be the initiator codon for the *atp8* and *nad1* genes. Ten PCGs ended with complete stop codon TAA, whereas the *nad4* gene was terminated by incomplete stop codon TA, and *cytb* and *nad5* were terminated by a single T. The presence of incomplete stop codons is common phenomenon in invertebrate mitochondrial genes, which is presumably completed as TAA via post‐transcriptional polyadenylation (Cannicci et al., 2017; Ivey & Santos, 2007; Ojala, Montoya, & Attardi, 1981).

The RSCU values for the 13 PCGs were summarized in Table 3. The *M. lauensis* and *M. verrilli* mitogenomes encoded 3,699 and 3,710 amino acids, respectively. The amino acids Ser (RSCU = 2.19), Leu (RSCU = 2.14), and Phe (RSCU = 1.62) were mostly used in *M. lauensis* mitogenome. Also in *M. verrilli* mitogenome, Ser (RSCU = 2.20), Leu (RSCU = 2.07), and Phe (RSCU = 1.55) were the most common amino acids. RSCU also reflects a nucleotide composition bias in *M. lauensis* and *M. verrilli* mitogenomes. The RSCU values for the codons NNU and NNA were usually higher than 1, suggesting a strong A+T‐bias in their third codon position (Table 3). This result supports the hypothesis that there should be a positive correlation between the codon usage bias and the AT bias of the third codon position for the mitogenomes (Chai, Du, & Zhai, 2012; Hao et al., 2012; Kim et al., 2009; Salvato, Simonato, Battisti, & Negrisolo, 2008).

**Table 3:** Codon usage of *Munidopsis lauensis* (*Ml*) and *M. verrilli* (*Mv*) PCGs

Amino acid	Codon	*Ml*	*Mv*	Amino acid	Codon	*Ml*	*Mv*
		*N* (RSCU)^a^	*N* (RSCU)^a^			*N* (RSCU)^a^	*N* (RSCU)^a^
F	UUU	261 (1.62)	260 (1.55)	Y	UAU	63 (1.00)	70 (1.12)
	UUC	62 (0.38)	75 (0.45)		UAC	63 (1.00)	55 (0.88)
L	UUA	206 (2.14)	211 (2.07)	H	CAU	14 (0.33)	29 (0.69)
	UUG	109 (1.13)	108 (1.06)		CAC	70 (1.67)	55 (1.31)
L	CUU	82 (0.85)	97 (0.95)	Q	CAA	55 (1.49)	61 (1.65)
	CUC	56 (0.58)	73 (0.72)		CAG	19 (0.51)	13 (0.35)
	CUA	99 (1.03)	98 (0.96)	N	AAU	62 (0.89)	80 (1.18)
	CUG	26 (0.27)	24 (0.24)		AAC	78 (1.11)	56 (0.82)
I	AUU	185 (1.34)	197 (1.34)	K	AAA	57 (1.31)	64 (1.35)
	AUC	92 (0.66)	97 (0.66)		AAG	30 (0.69)	31 (0.65)
M	AUA	104 (1.30)	116 (1.27)	D	GAU	28 (0.80)	27 (0.93)
	AUG	56 (0.70)	66 (0.73)		GAC	42 (1.20)	31 (1.07)
V	GUU	89 (1.30)	96 (1.51)	E	GAA	53 (1.15)	46 (1.06)
	GUC	35 (0.51)	27 (0.43)		GAG	39 (0.85)	41 (0.94)
	GUA	70 (1.02)	64 (1.01)	C	UGU	27 (1.15)	28 (1.22)
	GUG	80 (1.17)	67 (1.06)		UGC	20 (0.85)	18 (0.78)
S	UCU	93 (2.19)	99 (2.20)	W	UGA	56 (1.18)	60 (1.24)
	UCC	28 (0.66)	43 (0.96)		UGG	39 (0.82)	37 (0.76)
	UCA	48 (1.13)	49 (1.09)	R	CGU	10 (0.66)	12 (0.80)
	UCG	9 (0.21)	11 (0.24)		CGC	11 (0.72)	6 (0.40)
P	CCU	37 (1.00)	41 (1.12)		CGA	31 (2.03)	30 (2.00)
	CCC	59 (1.59)	54 (1.47)		CGG	9 (0.59)	12 (0.80)
	CCA	36 (0.97)	41 (1.12)	S	AGU	23 (0.54)	32 (0.71)
	CCG	16 (0.43)	11 (0.30)		AGC	35 (0.82)	20 (0.44)
T	ACU	43 (1.00)	62 (1.27)		AGA	40 (0.94)	56 (1.24)
	ACC	65 (1.51)	60 (1.23)		AGG	64 (1.51)	50 (1.11)
	ACA	54 (1.26)	57 (1.17)	G	GGU	47 (0.80)	44 (0.75)
	ACG	10 (0.23)	16 (0.33)		GGC	46 (0.79)	36 (0.61)
A	GCU	93 (1.56)	87 (1.47)		GGA	52 (0.89)	76 (1.29)
	GCC	77 (1.29)	75 (1.27)		GGG	89 (1.52)	79 (1.34)
	GCA	47 (0.79)	53 (0.90)				
	GCG	22 (0.37)	21 (0.36)				

Note

*N*: number of occurrence of the codon; RSCU, relative synonymous codon usage.

a The value in the brackets refer to the RSCU.

**Figure 2:**
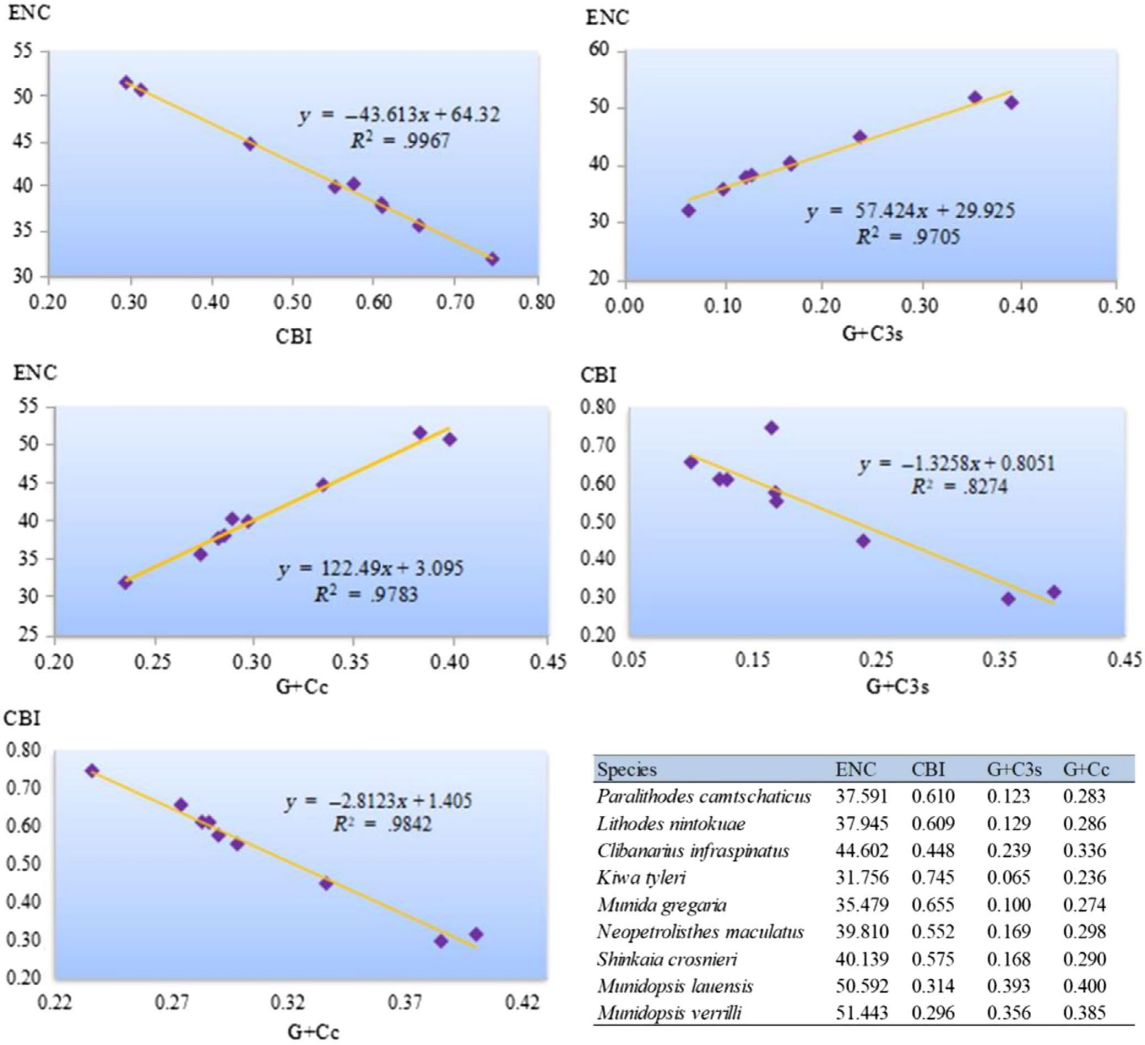
Evaluation of codon bias in the mitogenomes of twelve anomuran species. ENC, effective number of codons; CBI, codon bias index; G+Cc, G+C content of all codon positions; G+C3s, G+C content of the third codon positions

In order to further explore the codon usage bias among anomuran species, we analyzed the correlations between the effective number of codons (ENC), codon bias index (CBI), the G+C content of all codons (G+Cc), and the G+C content of the third codon position (G+C3s). We found ENC and CBI (*R* ^2^ = .997), CBI and G+Cc (*R* ^2^ = .984), and CBI and G+C3s (*R* ^2^ = .827) were negatively related, whereas ENC and G+Cc (*R* ^2^ = .978), and ENC and G+C3s (*R* ^2^ = .971) were positively related (Figure 2). These results are in consistent with the neutral mutational theories that the codon usage bias among organisms are mostly determined by the G+C content of the mitogenomes (Chen, Lee, Hottes, Shapiro, & McAdams, 2004; Plotkin & Kudla, 2011).

### 3.4 Transfer and ribosomal RNA genes

**Figure 3:**
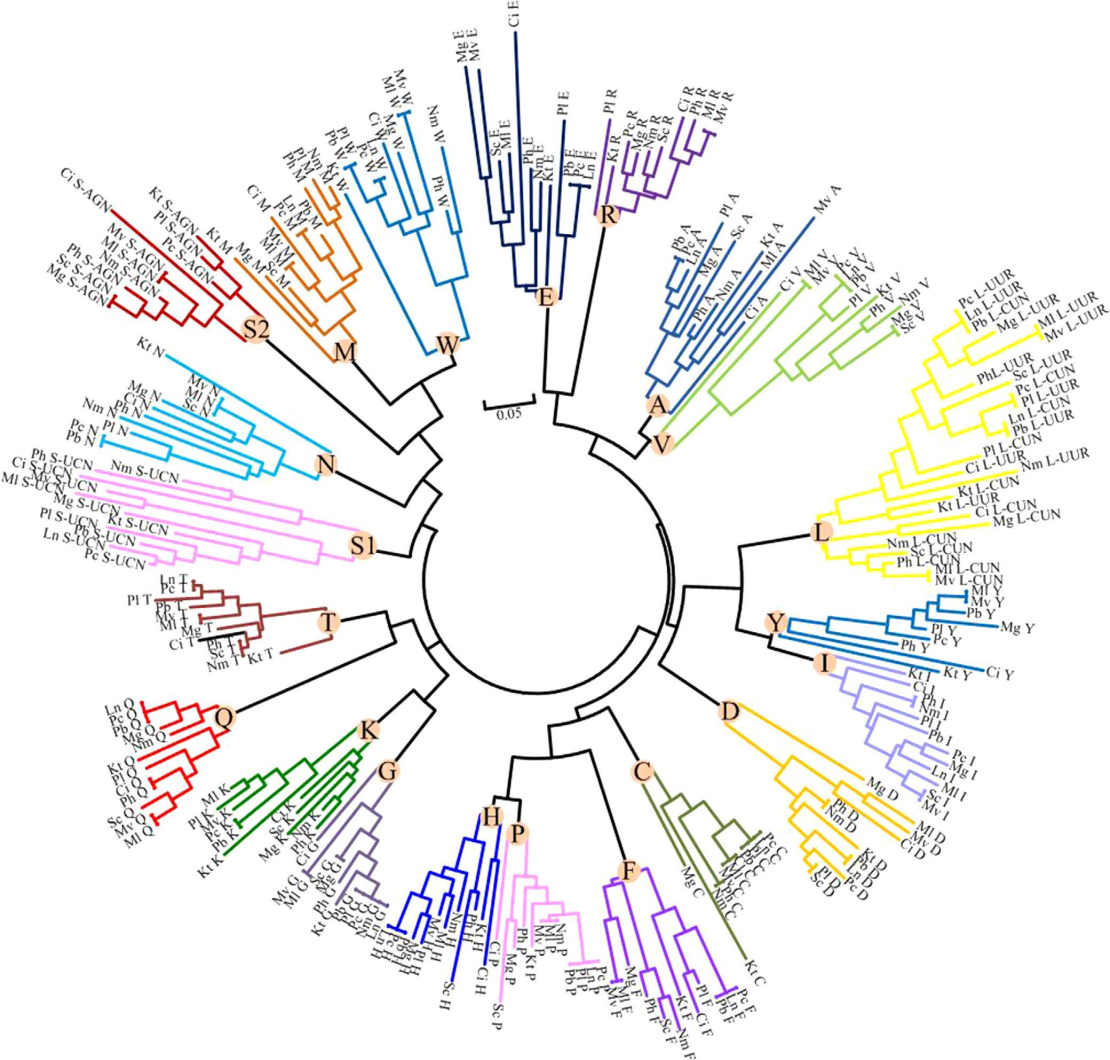
Neighbor‐joining tree based on uncorrected *p* distances among mitochondrial tRNA genes from twelve anomuran species. Pb, *Paralithodes brevipes*; Ph, *Petrolisthes haswelli*; Pl, *Pagurus longicarpus*; Pc, *Paralithodes camtschaticus*; Ln, *Lithodes nintokuae*; Ci, *Clibanarius infraspinatus*; Kt, *Kiwa tyleri*; Mg, *Munida gregaria*; Nm, *Neopetrolisthes maculatus*; Sc, *Shinkaia crosnieri*; Ml, *Munidopsis lauensis*; and Mv, *Munidopsis verrilli*

The complete set of 22 tRNA genes, typical of metazoan mitogenomes (two for each of serine and leucine, and one for each of the other 18 amino acids), were identified from in *M. lauensis* and *M. verrilli* mitogenomes. The tRNA genes ranged from 63 bp (*trnG*, as well as *trnY* in *M. verrilli* mitogenome) to 73 bp (*trnP*) in size and showed a strong A+T bias (69.59% and 69.25% in *M. lauensis* and *M. verrilli*, respectively). The AT‐skews were positive and GC‐skew were negative for the tRNA genes in both mitogenomes. Almost all of the tRNAs could be folded into a typical clover‐leaf secondary structures containing four functional arms and corresponding loops (Figures S1 and S2). However, *trnS*1 had no dihydrouridine (DHU) arm in the secondary structure. Although the tRNA content was conserved in *Munidopsis* mitogenomes, their arrangement was specific (see Section 3.5). The tRNA gene rearrangement in mitochondrial genomes can probably be explained by tandem duplication mechanism and tRNA gene recruitment (Dowton & Austin, 1999; Wang & Lavrov, 2011). In order to explore the possible evolutionary mechanism of tRNA gene rearrangement in *Munidopsis* mitogenomes, we analyzed tRNA gene sequences from 12 Anomura mitogenomes. The NJ tree showed that the equivalent tRNA genes (with the same amino acid and anticodon identities) from different species form well‐defined clades (Figure 3). This result revealed the orthologous relationships of each equivalent tRNAs. Thus, the tRNA gene rearrangement in *Munidopsis*, and even other anomuran mitogenomes, most probably arises from tandem duplication and random loss of tRNA genes, instead of tRNA gene recruitment.

The *rrnL* genes were located between *trnL_1_* and *trnV*, while *rrnS* were located between *trnV* and CR (Figure 1 and Table 1). In *M. lauensis* and *M. verrilli* mitogenomes, the A+T content of the two rRNA genes were 70.49% and 70.83%, respectively, which were the lowest among anomuran species (Table 2). The AT‐skew of the two rRNAs was negative (−0.015) in *M. lauensis*, while it was positive (0.002) in *M. verrilli*. The GC‐skew in both species were positive (0.384 and 0.386, respectively).

### 3.5 Control region

**Figure 4:**
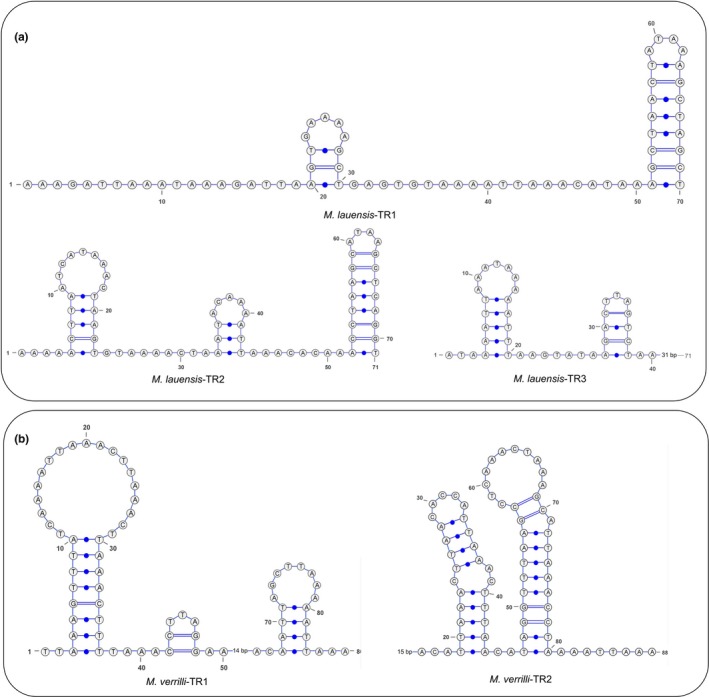
Stem‐loop structures of the tandem repeat motif in the control region of (a) *Munidopsis lauensis* and (b) *M. verrilli* mitogenomes

Twenty‐six noncoding regions, totaling 2,754 bp, were interspersed throughout the *M. lauensis* mitogenome, while the corresponding values were 24 and 2,875 bp in *M. verrilli*. The noncoding regions located between *rrnS* and *trnQ* (650 and 647 bp in *M. lauensis* and *M. verrilli*, respectively) corresponds to the CR identified in other decapods, which may contain the signals for replication and transcription (Taanman, 1999). The A+T content of the predicted control region in *M. lauensis* and *M. verrilli* was 73.38% and 75.27%, respectively, with both negative AT‐skew (0.392 and 0.228) and positive GC‐skew (−0.283 and −0.313). In the CR of *M. lauensis* mitogenome, one 205‐bp tandem repeat region (9,723–9,927) was found, which comprised three nearly identical motifs with 70, 71, and 64 bp in length, respectively (Figure 4). The CR of *M. verrilli* contained a 174‐bp repeat sequence (9,518–9,691), which included two nearly identical motifs (Figure 4). The slipped‐strand mispairing during mtDNA replication may result in the occurrence of tandem repeats (Levinson & Gutman, 1987). Each tandem repeat motif could be folded into stem‐loop secondary structures (Figure 4), which may play an important part in mtDNA duplications (Stanton, Daehler, Moritz, & Brown, 1994; Wilkinson & Chapman, 1991). Additionally, special “G(A)_n_T” motif and AT‐rich sequences were also observed in the CRs of *M. lauensis* and *M. verrilli*. Similar characteristics were also reported in the deep‐sea anemone *Bolocera* sp. (Zhang, Zhang, Wang, Zhang, & Lin, 2017), deep‐sea spongicolid shrimp *Spongiocaris panglao* (Sun et al., 2018a), and the hydrothermal vent alvinocaridid shrimp *Shinkaicaris leurokolos* (Sun, Hui, Wang, et al., 2018).

### 3.6 Mitochondrial gene order and rearrangements

**Figure 5:**
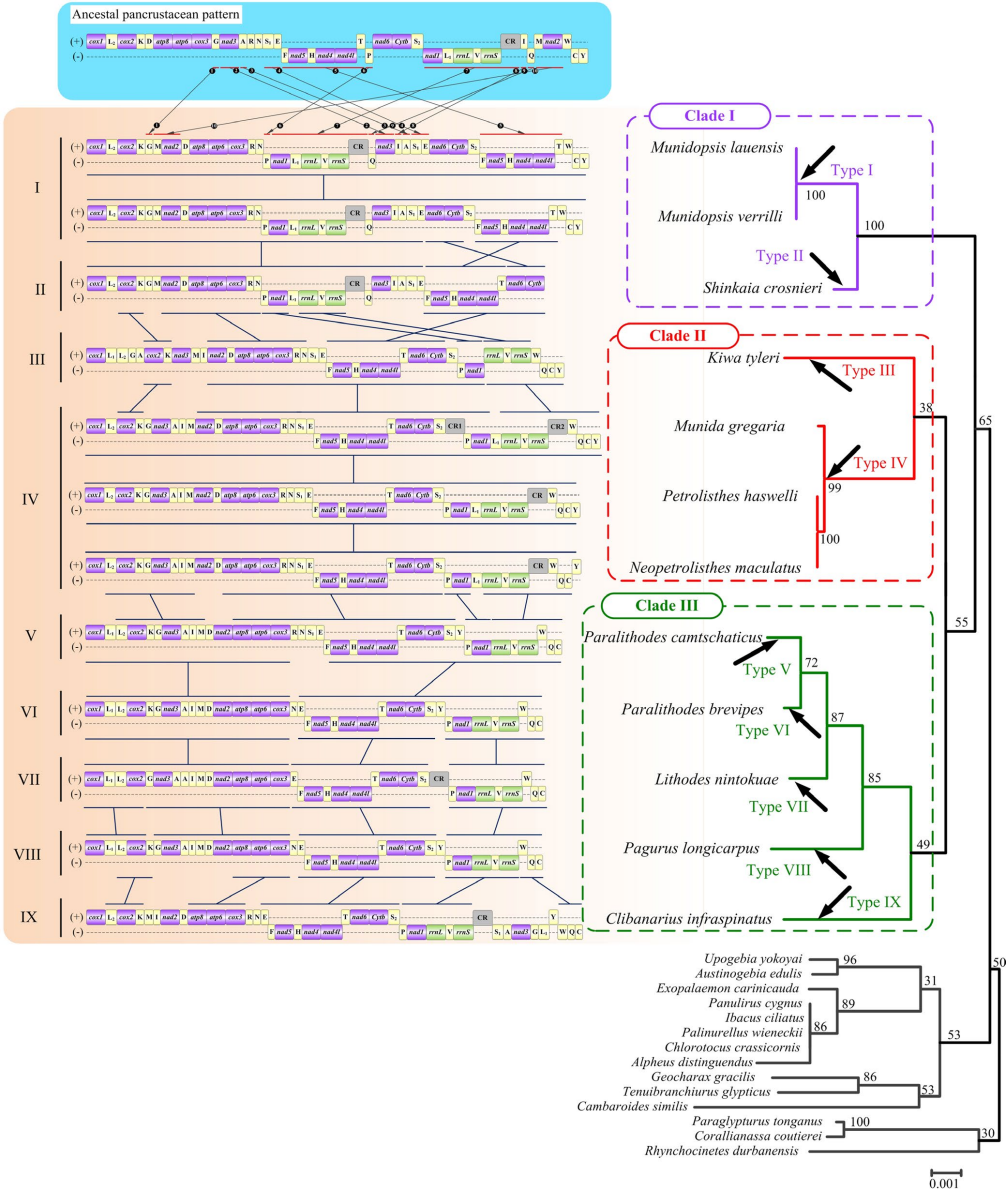
Phylogeny reconstructed by gene order, and arrangement of mitochondrial genes in the ancestral pancrustacean pattern and the infraorder Anomura. *Cox1* has been designated the start point for the linear representation of the gene arrangement. All genes are transcribed from left to right. The abbreviations of the genes are the same as Figure 1. The unassigned regions are not presented and gene segments are not drawn to scale. The bars indicate identical gene blocks

The *M. lauensis* and *M. verrilli* showed a novel arrangement of mitochondrial genes (Figure 5). Their gene order diverged in many positions from that of the ancestral pancrustacean pattern, which is shared by lots of crustaceans and hexapods (Boore, Lavrov, & Brown, 1998). Totally, we identified at least ten rearrangements in *M. lauensis* and *M. verrilli* mitogenomes compared with the ancestral pancrustacean pattern (Figure 5). The main rearrangements were tRNA translocations, and four rearrangements involved in PCGs. One of the major fragment containing *trnF*, *nad5*, *trnH*, *nad4*, *nad4l*, and *trnT* moved to downstream of *trnS_2_* from its ancestral position; the other major fragment containing *nad1*, *trnL_1_*, *rrnL*, *trnV*, *rrnS*, and CR moved to downstream of *trnN*. The *nad3* gene, located between *trnG* and *trnA*, translocated to the position between *trnQ* and *trnI*. And the fraction *trnM‐nad2* was located between *trnG* and *trnD* instead of the original position between *trnQ* and *trnW* genes. The *trnG*, *trnA*, *trnP*, *trnQ* moved to upstream of *trnM*, *trnS_1_*, *nad1*, *nad3*, respectively. The *trnI* moved to the downstream of *nad3* gene. The gene block *trnS1‐trnE* translocated to the middle of *trnA* and *nad6*. According to the CREx analyses, these novel gene orders of *M. lauensis* and *M. verrilli* might be the result of 3 tandem duplication–random loss (tdrl) events from the ancestral pancrustacean pattern (Figure S3).

**Figure 6:**
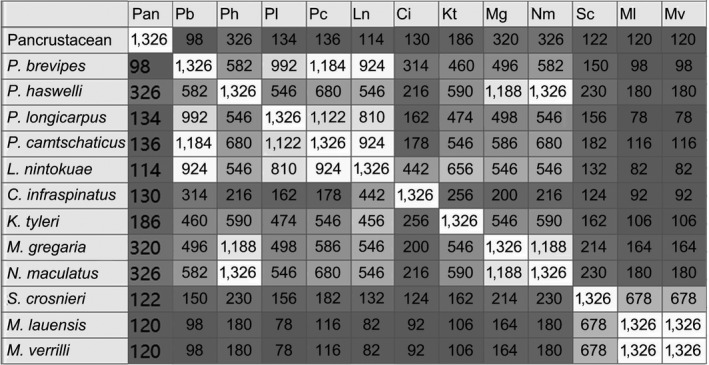
Pairwise comparisons of mitochondrial gene orders in anomurans obtained from CREx analysis. The numbers indicate the similarities of the compared gene orders, where 1,326 is the highest number and represents identical gene order

The twelve anomurans exhibited nine types of gene organization, which differ from any gene order ever reported in decapods. *P. haswelli*, *M. gregaria*, and *N. maculatus* showed the most similarities in mitochondrial gene order with the ancestral pancrustacean pattern (Figure 6). The mitochondrial gene orders of *M. lauensis* and *M. verrilli* (Type I in Figure 5) shared the most similarities with *S. crosnieri* (Type II). This result was consistent with previous study (Yang & Yang, 2008). *K. tyleri* (Type III) shared higher similarities with Type IV (*P. haswelli/M. gregaria/N. maculatus*. These results are consistent with the conclusion from the gene order‐based phylogenetic tree (Figure 5). *M. lauensis* and *M. verrilli* showed a closest relationship with *S. crosnieri* in the gene order tree (Clade I). *K. tyleri* clusters with the *P. haswelli/M. gregaria/N. maculatus* group (Clade II). The Clade III contained all other anomuran species. Our results support that comparisons of mitochondrial gene rearrangements, to some extent, are a useful tool for phylogenetic studies.

Comparative analysis of mitochondrial gene order has been proved to be a valuable phylogenetic tool in crustaceans (Shen, Tsang, Chu, Achituv, & Chan, 2015; Xin et al., 2017). Based on the comparative analysis of mitochondrial gene arrangement within Sessilia, Shen et al. (2015) found that *Amphibalanus amphitrite* (Balanidae) should cluster with *Striatobalanus amaryllis* (Archaeobalanidae) and *Nobia grandis* (Pyrgomatidae) instead of *Megabalanus* (Balanidae), resulting in nonmonophyly of the family Balanidae. Xin et al. (2017) examined the mitochondrial gene arrangements of infraorder Brachyura and suggested that *Clistocoeloma sinensis* may belong to the group Sesarmidae of the superfamily Grapsoidea and that *C. sinensis* and *Sesarmops sinensis* probably belong to sister groups.

### 3.7 Phylogenetic analysis

**Figure 7:**
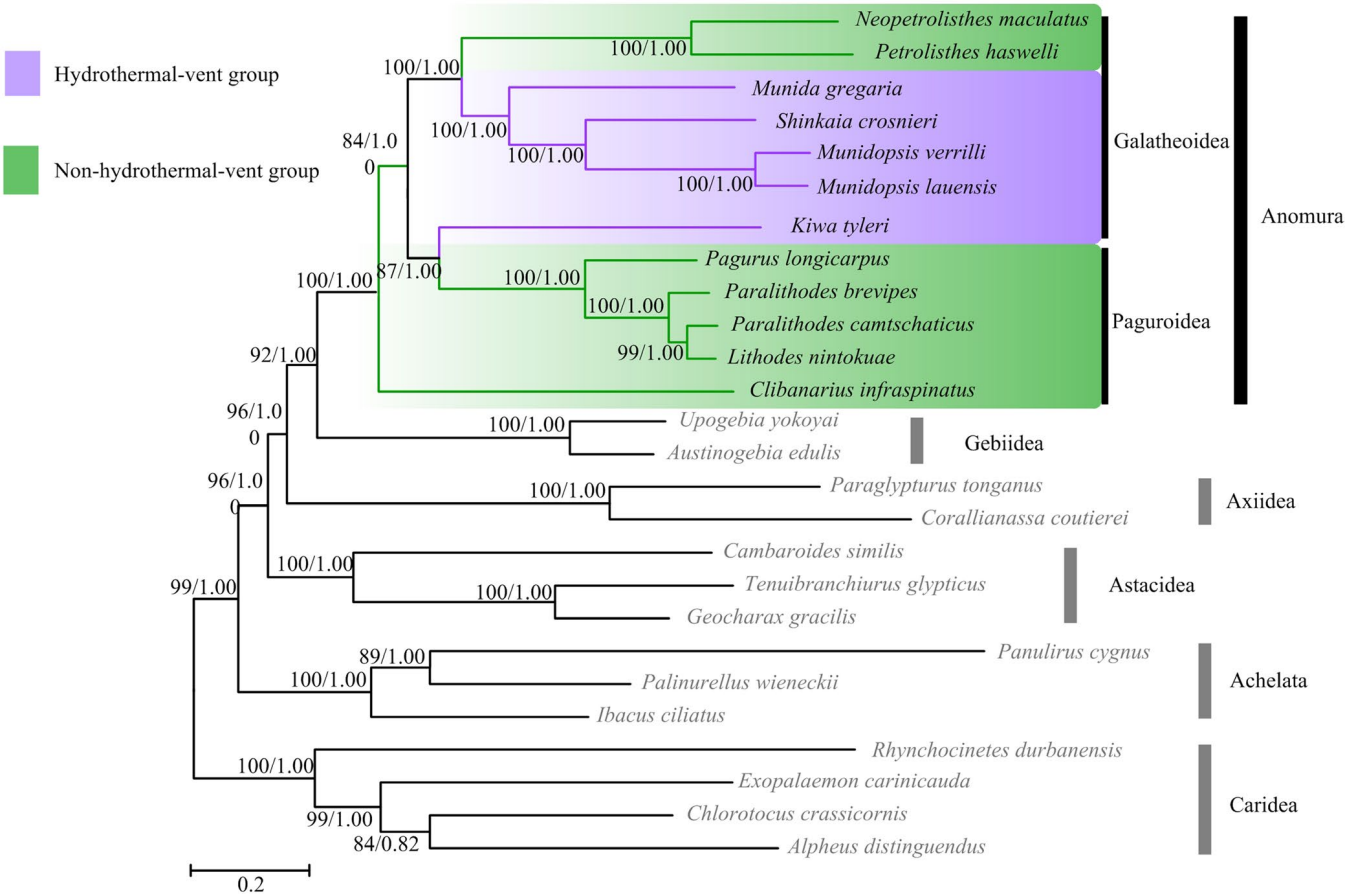
Phylogenetic trees derived from maximum likelihood and bayesian analyses based on Anomura mitochondrial PCGs and rRNA sequences with bootstrap values shown on branches. The first number at each node is the bootstrap probability of ML analyses and the second number is Bayesian posterior probability

Regardless of different inference methods (BI or ML), the two trees displayed identical topology with high nodal support values (Figure 7). The twelve anomuran species included in this analysis separated into three highly supported clades, one solely comprised of Paguroidea species, *C. infraspinatus*. The second group consisted of the remaining Paguroidea species and the hydrothermal vent yeti crab *K. tyleri* from Galatheoidea. Thus, traditional placement of *K. tyleri* within Galatheoidea based on morphology was not retrieved by our analyses, which is similar to the previous study based on molecular and morphological data (Schnabel et al., 2011). The third group contained all the remaining Galatheoidea species. Thus, the monophyly of the superfamily Paguroidea and Galatheoidea was not supported. Although the phylogeny of Anomura obtained from nucleotide sequences was inconsistent with that from gene order data, the closest relationship between the hydrothermal vent squat lobsters *M. lauensis*/*M. verrilli* and *S. crosnieri* was highly supported in both phylogenies.

Interestingly, the hydrothermal vent galatheid crabs were placed at more evolved positions in the trees. These observations suggested that they migrated from hydrothermal vent environments, instead of the remnants of ancient hydrothermal vent species, which support the extinction/repopulation hypothesis (Jacobs & Lindberg, 1998). This invasion event was also found in hydrothermal vent alvinocarid shrimps (Sun, Sha, & Wang, 2018b).

### 3.8 Positive selection analysis

In the analysis of branch‐specific models, the “two‐ratios” (M2) model did not fit the data significantly better than “one‐ratio” (M0) model when we set the vent anomurans as a foreground branch (*p* > .05, Table 4). LRTs based on the branch‐site models (MA vs. Null model) detected significant signals of positive selection in seven genes (*atp8*, *Cytb*, *nad3*, *nad4*, *nad4l*, *nad5*, *and nad6*) along the hydrothermal vent anomuran branches (Table 4). In total, eighteen positively selected residues were identified by the BEB analyses (BEB value >0.95).

**Table 4:** Selective pressure analyses of the mitochondrial genes of Anomura lineage

Branch model								
Models	LnL		Estimates of parameters			Model compared		2ΔL
Free‐ratio model (M1)	−63,224.5337					M1 versus M0		418.2264**
Two‐ratio model (M2)	−63,430.4760		*ω*0 = 0.04065 *ω*1 = 0.03082			M2 versus M0		6.3418
One‐ratio model (M0)	−63,433.6469		*ω* = 0.03951					

Branch‐site model										
Genes	Models	LnL	Estimates of parameters					Model compared	2ΔL	Positively selected sites (BEB analysis)
			Site class	0	1	2a	2b			
atp8	Model A	−1,308.57	Proportion	0.62298	0.28054	0.06653	0.02996	Model A versus Null model	6.388*	34 E (0.979)
			Background *ω*	0.0555	1.0000	0.0555	1.0000			
			Foreground *ω*	0.0555	1.0000	318.2622	318.2622			
	Null model	−1,311.76								
Cytb	Model A	−7,328.57	Proportion	0.9343	0.0269	0.0377	0.0011	Model A versus Null model	0.000	361 W (0.954)
			Background *ω*	0.0230	1.0000	0.0230	1.0000			
			Foreground *ω*	0.0230	1.0000	1.0000	1.0000			
	Null model	−7,328.57								
nad3	Model A	−2,405.14	Proportion	0.6570	0.0174	0.3171	0.0084	Model A versus Null model	0.500	15 S (0.997) 29 T (0.969) 32 D (0.997) 36 N (1.000) 38 P (0.997) 47 G (0.993) 72 T (0.993) 79 Y (0.950) 98 F (0.987)
			Background *ω*	0.0149	1.0000	0.0149	1.0000			
			Foreground *ω*	0.0149	1.0000	999.0000	999.0000			
	Null model	−2,405.39								
nad4	Model A	−8,916.40	Proportion	0.9056	0.0502	0.0419	0.0023	Model A versus Null model	3.735	83 P (0.998)
			Background *ω*	0.0209	1.0000	0.0209	1.0000			
			Foreground *ω*	0.0209	1.0000	35.3398	35.3398			
	Null model	−8,918.26								
nad4l	Model A	−1,949.71	Proportion	0.9023	0.0258	0.0699	0.0020	Model A versus Null model	1.630	43 C (0.970)
			Background *ω*	0.0225	1.0000	0.0225	1.0000			
			Foreground *ω*	0.0225	1.0000	57.3451	57.3451			
	Null model	−1,950.52								
nad5	Model A	−12,180.92	Proportion	0.8058	0.0998	0.0841	0.0104	Model A versus Null model	0.015	281 S (0.991) 398 S (0.995) 507 S (0.967)
			Background *ω*	0.0290	1.0000	0.0290	1.0000			
			Foreground *ω*	0.0290	1.0000	50.0604	50.0604			
	Null model	−12,180.93								
nad6	Model A	−4,149.03	Proportion	0.7962	0.1152	0.0774	0.0112	Model A versus Null model	9.889**	2 R (0.986) 123 A (0.999)
			Background *ω*	0.0336	1.0000	0.0336	1.0000			
			Foreground *ω*	0.0336	1.0000	57.6113	57.6113			
	Null model	−4,153.98								

** 0.001 < *p* < 0.01

* *p* < 0.001

The mitogenome is characterized by its adaptations to the extreme living environments (Castellana, Vicario, & Saccone, 2011). One major adaptation of galatheid squat lobsters is positive selection on mitochondrial genes involved in energy metabolism, hypoxia response, and sulfide‐tolerating. NADH dehydrogenase complex (Complex I), acting as a proton pump, is the first and the largest enzyme complex in the respiratory chain (Da Fonseca, Johnson, O'Brien, Ramos, & Antunes, 2008; Mishmar et al., 2003). Cytochrome *b* (Complexes III) use direct coupling for electron transfer and proton translocation (Sazanov, 2015). As part of the regulatory system of complex V (ATP synthase), *atp8* contribute to the proton translocation path and is directly associated with the produce of ATP (Anna et al., 2015; Castellana et al., 2011). These can to some extent explain why more positively selected sites were detected in complexes I, III, and V in our study. Similar results were found in hydrothermal vent alvinocaridid shrimps (Sun, Hui, Wang, et al., 2018; Wang et al., 2017), providing a better understanding of the adaptation of organisms to the deep‐sea vent environment.

## 4 CONCLUSIONS

In this study, we sequenced and annotated the complete mitogenomes of two squat lobsters *M. lauensis* and *M. verrilli* that colonized hydrothermal vents. Comparative mitogenomic analyses showed that gene content of the two mitogenomes was conserved, whereas gene arrangement displayed diversity. NJ analysis showed the tRNA rearrangements probably arise from tandem duplication and random loss of tRNA genes. CREx analyses reveal the most similarities of mitochondrial gene orders between *M. lauensis/M. verrilli* and *S. crosnieri*. The phylogenetic analyses based on both gene order data and nucleotide sequences (PCGs and rRNAs) also indicated that *M. lauensis* and *M. verrilli* were most closely related to *S. crosnieri*. Eighteen positively selected residues in seven genes (*atp8*, *Cytb*, *nad3*, *nad4*, *nad4l*, *nad5*, *and nad6*) were inferred to be positively selected sites for the branch of the hydrothermal vent anomurans, which may indicate that these genes experienced adaptive evolution.

## CONFLICT OF INTEREST

None declared.

## AUTHOR CONTRIBUTIONS

Sun S., Sha Z., and Wang Y. designed the manuscript, Sun S. and Sha Z. analyzed the data, and Sun S., Sha Z., and Wang Y. wrote the manuscript.

## Data Availability

DNA sequences: Genbank accession number MH717895 for *M. lauensis* and MH717896 for *M. verrilli*.
