# CORRIGENDUM

**DOI:** 10.1002/ece3.6890

**Published:** 2020-11-13

In “The complete mitochondrial genomes of two vent squat lobsters, *Munidopsis lauensis* and *M. verrilli*: Novel gene arrangements and phylogenetic implications”, which was published in volume 9 issue 22, November 2019, the authors discovered that there are errors in the first sentence of Section 2.1 | Sampling and DNA extraction. The depth and locations given for the hydrothermal vent chimney are incorrect. The corrected sentence and heading title for Section 2 can be found below:

## 2 METHODS

### 2.1 Sampling and DNA extraction

The hydrothermal vent squat lobsters, *M. lauensis* and *M. verrilli*, were captured from a hydrothermal vent chimney at a depth of 1374 m (122°41′59.326″E; 24°50′54.276″N) in the northwest Pacific Ocean.
