# Supplementary figures and images for: The complete mitochondrial genomes of two vent squat lobsters, Munidopsis lauensis and M. verrilli: Novel gene arrangements and phylogenetic implications
Source: Ecol Evol. 2019 Sep 30;9(22):12390–407. doi: 10.1002/ece3.5542 (PMC6875667; doi:10.1002/ece3.5542)

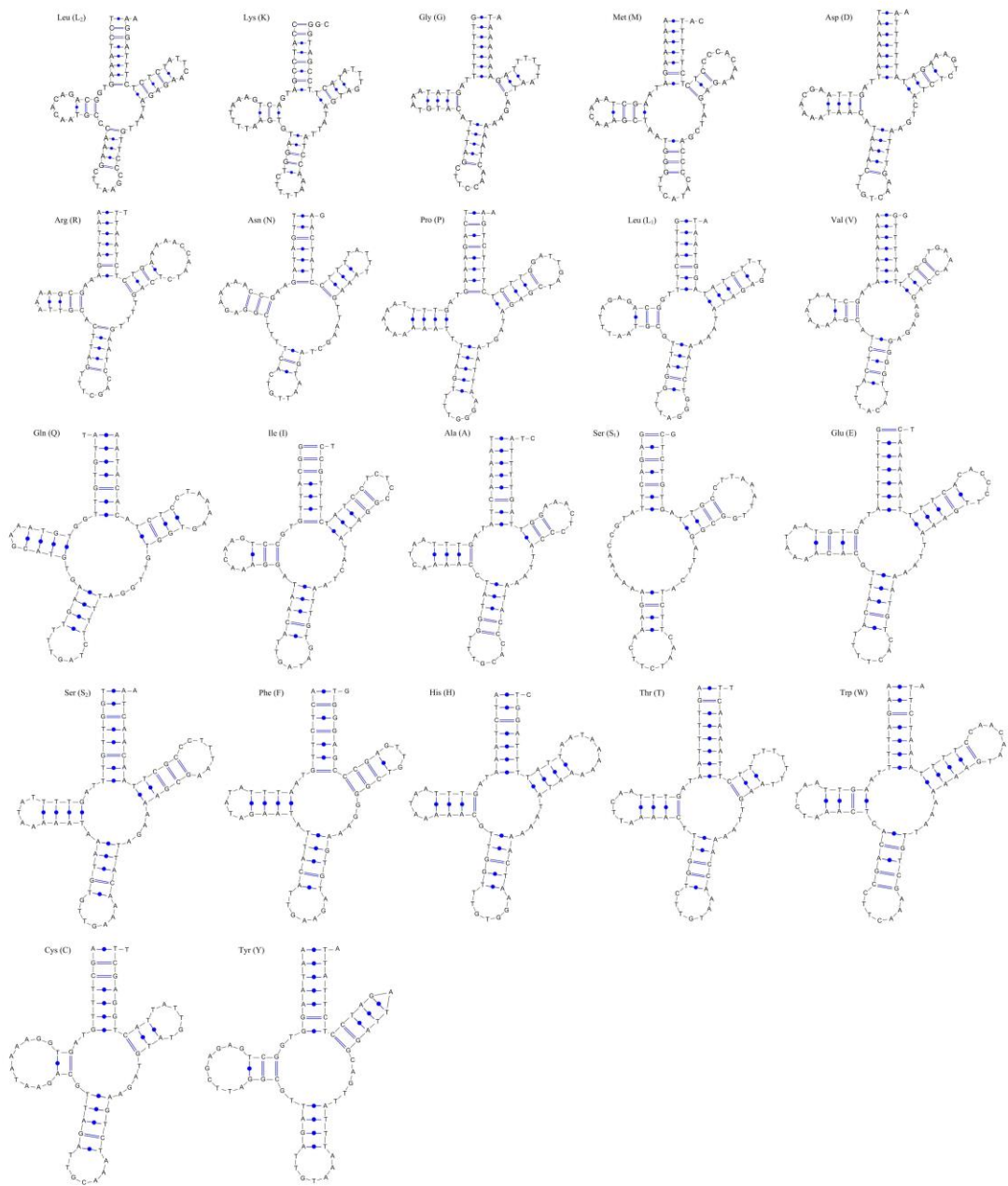

Supplement: Supplementary file 1 [file ECE3-9-12390-s001.pdf]

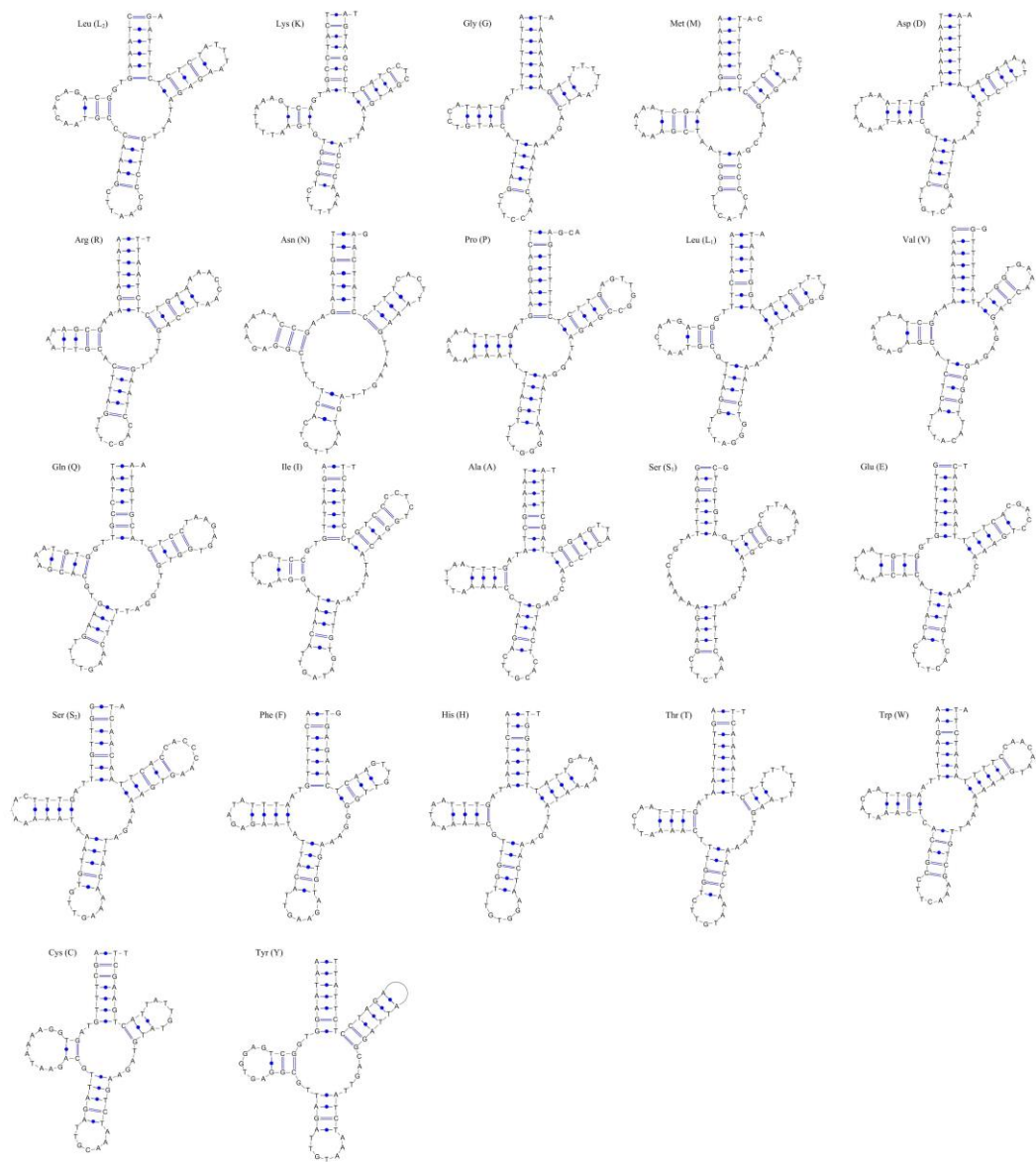

Supplement: Supplementary file 2 [file ECE3-9-12390-s002.pdf]
