# Supplementary material for: The complete mitochondrial genomes of two vent squat lobsters, Munidopsis lauensis and M. verrilli: Novel gene arrangements and phylogenetic implications
Source: Ecol Evol. 2019 Sep 30;9(22):12390–407. doi: 10.1002/ece3.5542 (PMC6875667; doi:10.1002/ece3.5542)
Supplement: Supplementary file 3 [file ECE3-9-12390-s003.doc]

**Supporting information Table 1** The species used in phylogenetic analysis.

| Species | GenBank NO. | Classification |  |
| --- | --- | --- | --- |
| *Paralithodes brevipes* | AB735677 | Anomura; Paguroidea; Lithodidae; Paralithodes | ingroup |
| *Petrolisthes haswelli* | LN624374 | Anomura; Galatheoidea; Porcellanidae; Petrolisthes | ingroup |
| *Pagurus longicarpus* | AF150756 | Anomura; Paguroidea; Paguridae; Pagurus | ingroup |
| *Paralithodes camtschaticus* | JX944381 | Anomura; Paguroidea; Lithodidae; Paralithodes | ingroup |
| *Lithodes nintokuae* | AB769476 | Anomura; Paguroidea; Lithodidae; Lithodes | ingroup |
| *Clibanarius infraspinatus* | LN626968 | Anomura; Paguroidea; Diogenidae; Clibanarius | ingroup |
| *Kiwa tyleri* | KY423514 | Anomura; Galatheoidea; Kiwaidae; Kiwa | ingroup |
| *Munida gregaria* | KU521508 | Anomura; Galatheoidea; Munididae; Munida | ingroup |
| *Neopetrolisthes maculatus* | KC107816 | Anomura; Galatheoidea; Porcellanidae; Neopetrolisthes | ingroup |
| *Shinkaia crosnieri* | EU420129 | Anomura; Galatheoidea; Galatheidae; Shinkaia | ingroup |
| *Munidopsis lauensis* | MH717895 | Anomura; Galatheoidea; Munidopsidae | ingroup |
| *Munidopsis verrilli* | MH717896 | Anomura; Galatheoidea; Munidopsidae | ingroup |
| *Ibacus ciliatus* | KM488334 | Achelata; Palinuroidea; Scyllaridae; Ibacus | outgroup |
| *Palinurellus wieneckii* | KC847078 | Achelata; Palinuroidea; Synaxidae; Palinurellus | outgroup |
| *Panulirus cygnus* | KT696496 | Achelata; Palinuroidea; Palinuridae; Panulirus | outgroup |
| *Geocharax gracilis* | HG942174 | Astacidea; Parastacoidea; Parastacidae; Geocharax | outgroup |
| *Tenuibranchiurus glypticus* | KM453741 | Astacidea; Parastacoidea; Parastacidae; Tenuibranchiurus | outgroup |
| *Cambaroides similis* | JN991196 | Astacidea; Astacoidea; Cambaridae; Cambaroides | outgroup |
| *Corallianassa coutierei* | KC107817 | Axiidea; Callianassidae; Corallianassa | outgroup |
| *Paraglypturus tonganus* | KJ820739 | Axiidea; Callianassidae; Paraglypturus | outgroup |
| *Alpheus distinguendus* | GQ892049 | Caridea; Alpheoidea; Alpheidae | outgroup |
| *Chlorotocus crassicornis* | KY944589 | Caridea; Pandaloidea; Pandalidae | outgroup |
| *Exopalaemon carinicauda* | EF560650 | Caridea; Palaemonoidea; Palaemonidae | outgroup |
| *Rhynchocinetes durbanensis* | KT590405 | Caridea; Nematocarcinoidea; Rhynchocinetidae | outgroup |
| *Upogebia yokoyai* | KM886610 | Gebiidea; Upogebiidae; Upogebia | outgroup |
| *Austinogebia edulis* | JN897376 | Gebiidea; Upogebiidae; Austinogebia | outgroup |
