# Supplementary material for: The complete mitochondrial genomes of two vent squat lobsters, Munidopsis lauensis and M. verrilli: Novel gene arrangements and phylogenetic implications
Source: Ecol Evol. 2019 Sep 30;9(22):12390–407. doi: 10.1002/ece3.5542 (PMC6875667; doi:10.1002/ece3.5542)
Supplement: Supplementary file 4 [file ECE3-9-12390-s004.doc]

**Supporting information Table 2** The best-fit partitioning schemes selected by PartitionFinder

| Partitions | Subset Partition | Best Model |
| --- | --- | --- |
| Partition 1 | *atp6_p1, nad3_p1, nad6_p1* | GTR+I+G |
| Partition 2 | *atp6_p2, cytb_p2, cox1_p2, cox2_p2, cox3_p2, nad1_p2, nad3_p2, nad4l_p2, nad4_p2, nad5_p2, nad6_p2* | GTR+I+G |
| Partition 3 | *atp6_p3, cytb_p3, cox1_p3, cox2_p3, cox3_p3, nad3_p3, nad6_p3* | HKY+G |
| Partition 4 | *rrnS*, *rrnL* | GTR+G |
| Partition 5 | *cytb_p1, cox1_p1, cox2_p1, cox3_p1* | GTR+I+G |
| Partition 6 | *nad1_p1, nad4l_p1, nad4_p1, nad5_p1* | GTR+I+G |
| Partition 7 | *nad1_p3, nad4l_p3, nad4_p3, nad5_p3* | GTR+G |
