# Supplementary material for: The complete mitochondrial genomes of two vent squat lobsters, Munidopsis lauensis and M. verrilli: Novel gene arrangements and phylogenetic implications
Source: Ecol Evol. 2019 Sep 30;9(22):12390–407. doi: 10.1002/ece3.5542 (PMC6875667; doi:10.1002/ece3.5542)

Ancestral pancrustacean pattern → Munidopsis

- family diagram for Ancestral pancrustacean pattern (e)

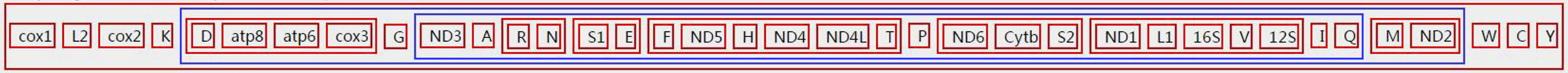

- family diagram for Munidopsis (e)

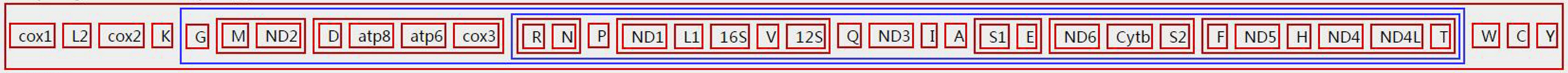

- scenario:
  - 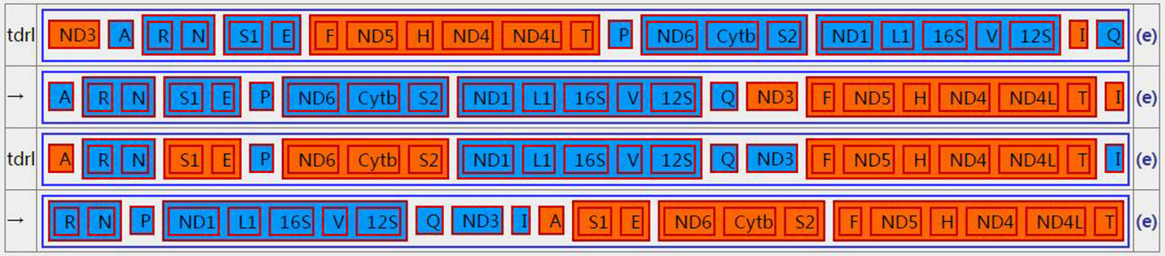

  - tdrl

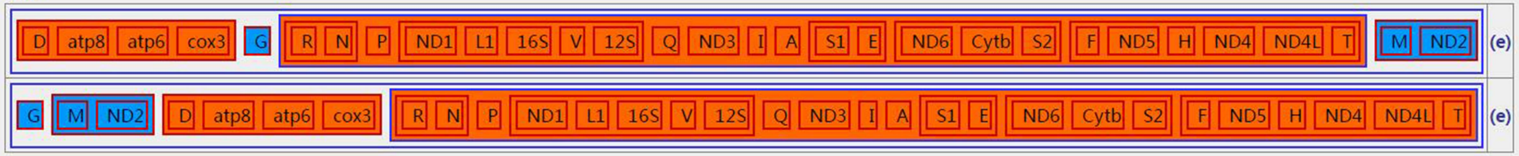

Supplement: Supplementary file 7 [file ECE3-9-12390-s007.pdf]
